# Toxicological Assessment of Trace β-Diketone Antibiotic Mixtures on Zebrafish (*Danio rerio*) by Proteomic Analysis

**DOI:** 10.1371/journal.pone.0102731

**Published:** 2014-07-25

**Authors:** Xiaohan Yin, Huili Wang, Yuna Zhang, Randy A. Dahlgren, Hongqin Zhang, Mengru Shi, Ming Gao, Xuedong Wang

**Affiliations:** 1 Institute of Wenzhou Applied Technology for Environmental Research, Wenzhou Medical University, Wenzhou, China; 2 Key Laboratory of Wenzhou Marine Biology, College of Life Sciences, Wenzhou Medical University, Wenzhou, China; Mayo Clinic, United States of America

## Abstract

β-Diketone antibiotics (DKAs) can produce chronic toxicity in aquatic ecosystems due to their pseudo-persistent in the environment. In this study, after long-term DKA exposure to zebrafish (*Danio rerio*), 47 protein spots had greater than 2-fold differential expression as compared to the control; there were 26 positive proteins with 14 up-regulated and 12 down-regulated. The main functions of the differentially expressed proteins were related to signal transduction mechanisms and the cytoskeleton. Of the 26 target genes, 11 genes were consistent between their transcriptional and translational levels. Low dose DKA exposure (4.69 and 9.38 mg/L) stimulated spontaneous movement in zebrafish. Changes in both creatine kinase activity and creatine concentration showed a similar trend to zebrafish activity. There was no obvious change in SV-BA after DKA exposure, while a reduction of heart rate was concomitant with increasing DKA concentrations. DKAs also induced severe histopathological changes in zebrafish heart tissue, such as dissolution of cristae and vacuolation of mitochondria. These results demonstrated that trace-level DKA exposure affects a variety of cellular and biological processes in zebrafish.

## Introduction

Fluoroquinolones and tetracyclines are two representative classes of widely used β-diketone antibiotics (DKAs). In recent years, DKAs have been extensively used in humans and livestock [1]. Because they are poorly absorbed in humans and livestock, the majority of the ingested compound is excreted to the environment in feces and urine. The large dose and frequent application of DKAs result in “pseudo-persistent” in the environment, even though half-lives of most DKAs species are relatively short – about 8 hours [2]–[3]. As a result, the activities of DKAs and their main metabolites can persist for a long time in aqueous environments. The residues of DKAs are detected extensively as exogenous environmental pollutants in soils, surface waters, and biological samples. Some DKAs, such as tetracycline, are easily washed into surface waters from soils by surface runoff [4].

DKAs can lead to environmental biological resistance, which contributes to severe liver and kidney toxicity and neurotoxicity. For example, acetylcholine esterase (AChE) activity was severely depressed in muscles of catfish exposed to enorxacin, which suggests a potential neurotoxic effect of enorxacin in fish [5]. Similarly, Hall and coworkers reported that fluoroquinolone antibiotics were associated with a wide array of musculoskeletal complications in tendon, cartilage, bone, and muscle tissues [6]. In detoxification metabolism, DKAs can induce glutathione (GSH) production at the end of the hatching period, as well as inhibit superoxide dismutase (SOD) activities [7]. Many previous studies have shown that DKAs have strong gene and genetic toxicity. For example, they may cause DNA damage, adduct formation, DNA hypermethylation in kidney cells of *Carassius auratus*, and synchronous teratogenic effects on *Cyprinus carpio* [8]–[9]. To date, most previous research has focused on the toxicity and relative mechanist effects for single DKA compound [7]–[9]; few studies have addressed exposure to mixtures of DKA compounds. In real-world environments, the joint action of different pollutants may be very complex. For example, perfluorooctane sulfonate (PFOS) and perfluorooctanoic acid (PFOA) showed interactive effects ranging from additive to synergistic [10] and Melvin and coworkers demonstrated increased toxicity (loss of tactile response) in striped marsh frog (*Limnodynastes peronii*) tadpoles exposed to a mixture of naproxen, carbamazepine, and sulfamethoxazole, compared to exposures to the individual compounds [11]. Hundreds of DKAs are often detected simultaneously in the environment and thus it is necessary to study the chronic toxicological effects of mixed DKA exposure in order to determine their integrated ecological risk.

Based on previous work with biomarkers, morphological development, and biological behavior of zebrafish under mixed DKA stress [7], we selected representative DKA species forexposure to zebrafish at the mg/L level, and analyzed zebrafish protein expression using 2-dimensional gel electrophoresis (2-DE) and MALDI-TOF-MS techniques. Additionally, the qRT-PCR technique was used to validate consistency of the 26 target genes between transcriptional and translational levels. Finally, the chronic toxicological molecular mechanisms associated with DKA exposure to zebrafish were determined based on: (1) proteomic analyses; (2) behavior activities; (3) enzymatic activity indices; and (4) histopathological analysis of cardiac tissue. These results provide the foundation for establishing a systematic toxicology model, showing their ecological risk, and diagnosing and treating the source of disease.

## Experimental

### Ethics statement

We confirm that the Institutional Animal Care and Use Committee (IACUC) at Wenzhou Medical University has approved our study plan for proper use of zebrafish. All studies were carried out in strict accordance to the guidelines of the IACUC. In the study of protein extraction, biomarker assay and histopathological analysis, a subset of the oldest zebrafish at three months were sacrificed for detailed study. All dissection was performed on ice, and all efforts were made to minimize suffering.

### Test chemicals

**Figure 1:**
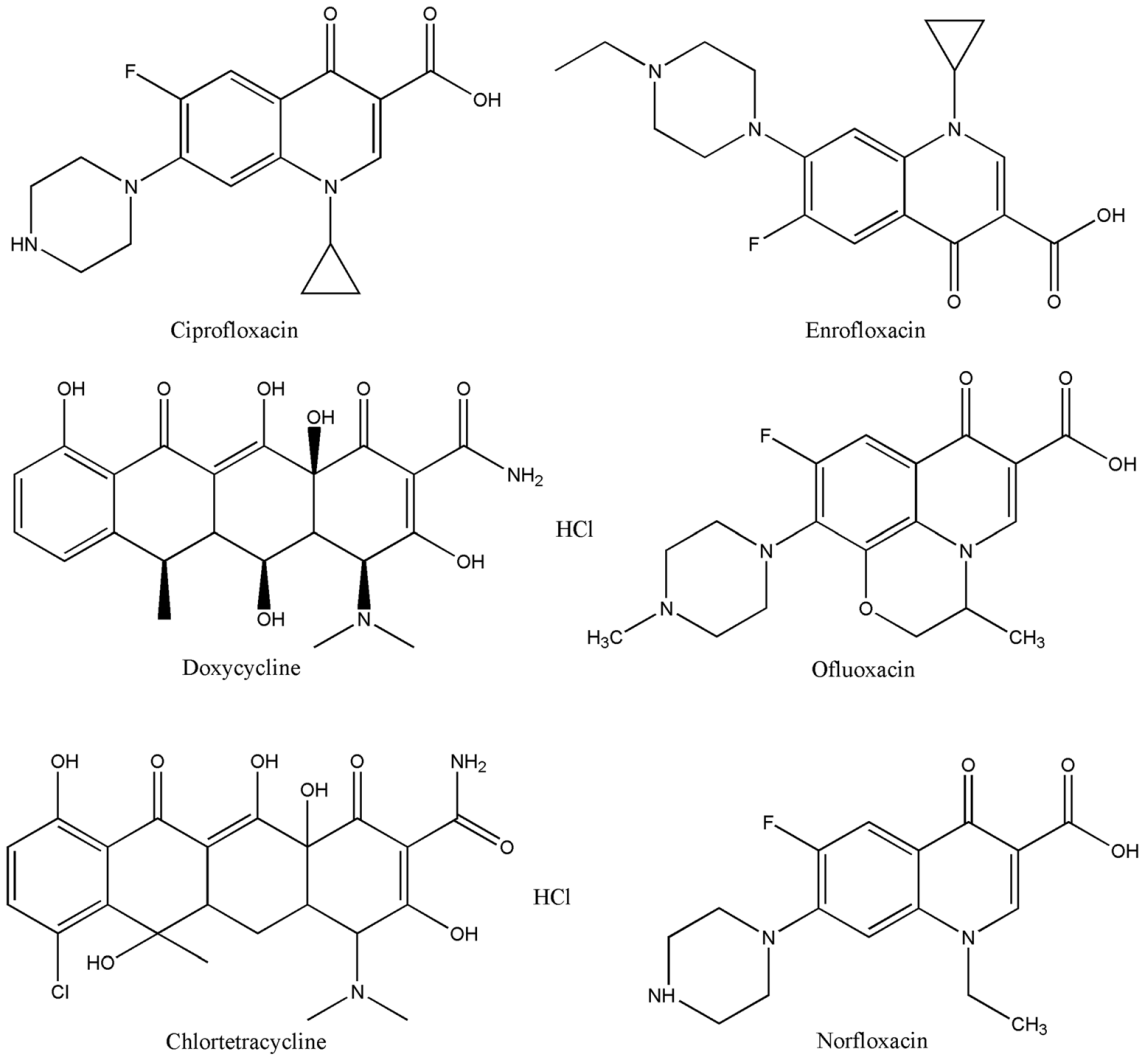
Chemical structures for each DKA compound.

Six representative DKAs were selected for study: ciprofloxacin (CAS No. 85721-33-1, 99%), ofluoxacin (CAS No. 82419-36-1, 99%), norfloxacin (CAS No. 70458-96-7, 99%), chlortetracycline (CAS No. 64-72-2, 95%), enrofloxacin (CAS No. 93106-60-6, 99%), and doxycycline (CAS No. 24390-14-5, 99%). The chemical structures for each DKA compound are shown in Fig. 1.

### Exposure experiment and sample preparation

Wild-type (AB strain) zebrafish were obtained from Oregon State University, USA, and raised according to guidelines for zebrafish cultivation [12]. Zebrafish were handled in compliance with animal welfare regulations and maintained according to standard protocols (http://ZFIN.org). DKA exposure was initiated at 6 hours post fertilization (hpf) with embryo medium (EM) for control and a 9.38 mg/L mixture of DKAs for treatment. The total exposure concentration for the six DKAs was selected according to previous studies that mixed equal weight concentrations and volumes of each antibiotic [7]. The embryos were cultivated and exposed until 144 hpf, and at this stage all embryos were hatched and survived without malformation. Then, zebrafish larvae were transferred to 2 L tanks for 6–30 days post fertilization (dpf). After 30 dpf, they were raised in 12 L tanks through the end of the experiment at 90 dpf. Control fish were raised identical to DKA treated fish with the exception of DKA exposure. The DKA treatment solution was renewed with freshly prepared solutions each day to ensure constant concentration throughout the duration of the experimental treatment. In order to ensure accuracy and reproducibility of the results, a minimum survival rate of greater than 95% was set for the control group.

### Protein extraction and two-dimensional gel electrophoresis (2-DE)

Protein extraction and quantification were performed following Bradford [13]. Zebrafish were homogenized by grinding in lysis buffer (9 M urea, 2 M thiourea, 0.1 M Tris-HCl, 4% CHAPS, 100 mM DTT, 1% Protease Inhibitor Cocktail) on ice. The 2-DE methodology followed our group's standard protocols with some modification [14]. In a preliminary experiment, the protein isoelectric points (pI) for most zebrafish were found to be pH 4–7 as evaluated by drystrips in the pH range of 3–10. Therefore, the maximum separation was obtained using pH 4–7 drystrips. To improve the dynamic range of protein quantification in silver-stained gel for visualization of both high- and low-abundance proteins on a 2-DE gel, pre-processing of images was performed as described by Grove et al. [15]. The protein spots were scanned using UNAX Powerlook 2100XL (Bio-Rad) and analyzed by ImageMaster 2D platinum 5.0 (Amersham Bioscience) according to manufacturer's instructions. Over the course of the entire experiment, three biological and three technological replicates yielded 9 gels.

### Image acquisition and data analysis

Image acquisition was conducted according to Wang et al. [14], [16]. The percentage volumes were used to designate significant differential expression spots (at least two-fold increase/decrease and statistical significance as calculated by the RVM-T test with a cutoff *p*-value <0.05 and FDR<0.05). Triplicate gels were used for each sample. Only those with reproducible and significant changes were considered to be differentially expressed protein spots. Data were reported as mean ± standard deviation (mean ± SD), which were calculated by SPSS 16.0 software (SPSS, Chicago, IL, USA). Differentially expressed protein spots were identified using the criterion >2-fold up-regulation or down-regulation.

### Protein identification by peptide mass fingerprinting (PMF) and MALDI-TOF-MS analysis

The differentially expressed protein spots were manually selected and excised, and then the gels were rehydrated and digested according to Wang et al. [16]. The peptide samples were analyzed with a MALDI-TOF-MS Proteomics Analyzer (Bruker Daltonics, Bremen, Germany). Data were obtained from the Internet using a Mascot search engine (Matrix Science Ltd., London, UK) against all entries in the NCBInr database. Only the significant hits, as defined by a MASCOT probability analysis (*p*<0.05), were accepted. If the theoretical values for MW and pI, which were identified by peptide mass fingerprinting (PMF) and MALDI-TOF-MS analysis, were consistent with the values determined by ImageMaster 2D platinum 5.0 in gel, we regarded the protein spot as a positive match. The matched peptides of this protein were blasted to the protein database nr (ftp://ftp.ncbi.nih.gov/blast/db/) and Swiss-Prot (ftp://ftp.uniprot. org/pub/databases/uniprot_datafiles_by_format/fasta/) in order to obtain the protein functional annotation information with the highest sequence similarity. Then, we acquired information on the ortholog of gene products and classification of gene functions by COG (ftp://ftp.ncbi.nih.gov/pub/COG/) functional classification. Differentially expressed proteins were grouped using COG online software.

### qRT-PCR analyses of the differentially expressed genes

Primer sets were designed using Primer Premier 5.0 software and synthesized by Shanghai Sangon Biotechnology Company (Shanghai, China) (Table S1). To validate the differentially expressed proteins of zebrafish at the transcriptional level *in vivo*, qRT-PCR was performed. Zebrafish at 90 dpf were used in the control and treatment groups. Total RNA was extracted and measured by the Nanodrop method to meet the requirements: OD_260_/OD_280_>1.8 and OD_260_/OD_230_>1.5. Then, cDNA was obtained by reverse transcription using a PrimeScript RT-PCR Kit with three technical replicates. All of the melting curves showed a single peak in the tested range of standard curves, indicating good specificity. No detectable fluorescence signal was observed in the negative and blank controls indicating that the reaction system was not contaminated during the experimental procedures. Relative quantification of the targets in each sample was carried out using the signal of β-actin as a stable reference gene.

### Effects of mixed DKA compounds on behavior of zebrafish larvae

A series of mixed DKA concentrations (0, 4.69, 9.38, 18.75, and 37.5 mg/L DKAs) were selected. These DKA concentrations had no obvious teratogenic effect on zebrafish larvae during the duration of the experimental process. DKA exposure time ranged from 6 to 96 hpf followed by a one-day detoxification and behavior monitoring beginning at 120 hpf. The larvae swimming speed was analyzed in a ZebraLab behavior monitoring station using a 24-well plate (Version 3.5 with background subtraction, Viewpoint Life Science, France) after the fixed habituation period. The light-to-dark stimulation responses of zebrafish were investigated, starting from darkness (10 min) and followed by two cycles of light (10 min) and dark (10 min). During this process, no visible zebrafish developmental malformation was observed from the DKA exposure.

### Biomarker assay

Zebrafish were exposed to different concentrations of DKAs (9.38, 45 and 60 mg/L) from 6 hpf to 90 dpf for the treatment group. Three biological replicates were used in each control and each DKA exposure treatment. Each control and treatment group at 90 dpf included 30 zebrafish. Fifteen fish were used for determination of creatine kinase (CK) and creatinine (Cr) concentrations for each 7-day period starting from the second week after DKA exposure. Cr concentration was determined in serum, and CK activity was measured in the supernatant after homogenizing the heart and muscle tissues. Blood was collected using a capillary tube after resection of the zebrafish tail, and then a moderate level of heparin was added to prevent blood clotting. Subsequently, the blood sample was centrifuged at 12,000 rpm for 10 min at 4°C to eliminate erythrocytes. Heart and muscle tissues were homogenized in a hand-held glass homogenizer on ice. The supernatant was collected for determination of CK activity. CK activity (U mg.prot^−1^) and Cr (µmol L^−1^) concentration were determined following manufacturer's specifications for test kits (Nanjing Jincheng Bioengineering Institute, Nanjing, China).

### Effects of mixed DKAs on cardiac development and heart rate (HR)

Well-developed oosperm were exposed to a series of DKA concentrations (0, 9.38, 18.75, 37.5, 75 and 150 mg/L) from 6 to 72 hpf. Zebrafish were put on ice throughout the experimental procedure to avoid movement; no anesthetic was used to ensure the accuracy and reliability of the experimental results. The solution for each treatment with three biological replicates was replaced once each day to maintain the desired DKA exposure concentration. 1-Phenyl-2-thiourea (PTU, 100 µM) was added into the solution for 20-hpf zebrafish to prevent the formation of melanin and to increase transparency for observation of cardiac development and measurement of HR. The distance between the sinus venosus and bulbus arteriosus (SV-BA) was determined using an inverted microscope, and the HR (atrium beats) was recorded 3 times over a 10 s period (mean ± SD) using a rapid DVC at 48, 60 and 72 hpf. Data were processed using Image J software to determine the heart rate. The heartbeat records of zebrafish were carried out at 28±0.5°C after a 5 min adaptation. Fifteen embryos for each treatment concentration were selected randomly, and the assay of all experimental groups was completed within 10 min.

### Histopathological analysis

Similar to the biomarker assay, DKA concentrations was set to 9.38, 45, 60, and 90 mg/L; these DKA concentrations resulted in no obvious malformation of zebrafish during the experimental process. DKA exposure occurred from 6 hpf to 90 dpf, and the three-month-old zebrafish were simultaneously used for both histopathological observation and proteomics study. After analyzing the anatomy of zebrafish in control and treatment groups, the heart was removed, cut into 1 mm^3^ tissue blocks, and fixed in pre-cooled glutaraldehyde at 4°C. Then, a series of pretreatment procedures, such as poaching, fixing, dehydrating, embedding, and sectioning, were carried out according to Sendzik et al. [17]. The treated tissue samples were observed using transmission electron microscope.

## Results

### 2-DE of differentially expressed proteins and MALDI-TOF MS analysis

**Figure 2:**
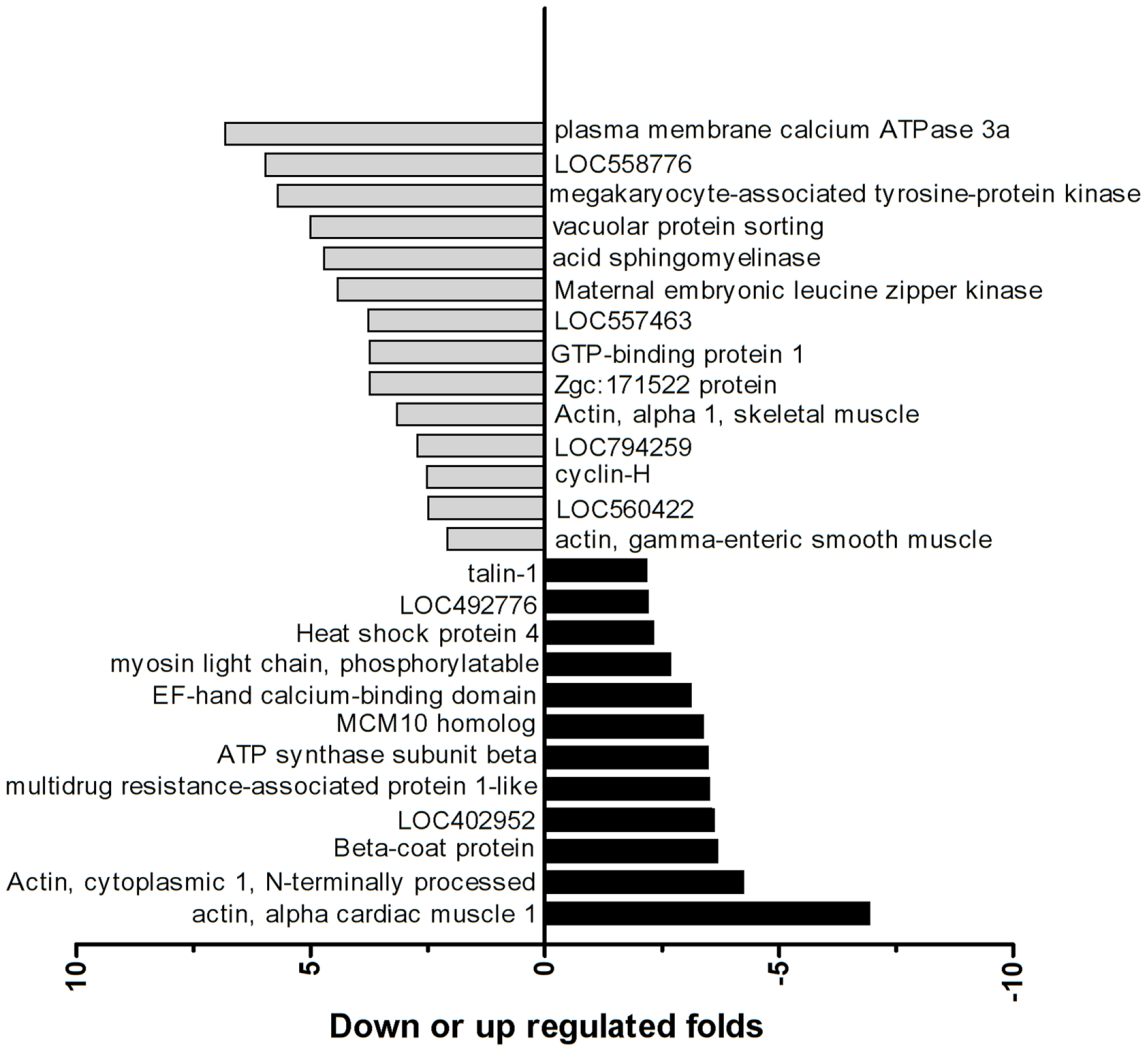
The differential protein spots between the DKA treatments and control groups. Note: The differential protein spots had >2.0-fold changes in the DKA treatment groups compared to the control group.

Previous reports of acute and chronic toxicities from single DKA species exposure found a wide range of effects on all kinds of target organs. However, DKAs may interact with each other and alter the toxicity of each individual species, with the combined toxicity being synergistic, antagonistic or additive. Because we did not know the main action target organ for mixed DKA exposure, proteomics analysis was performed with whole fish rather than one tissue or organ. Forty-seven protein spots were found to have greater than 2-fold differential expression compared to the control, and no protein spots were solely visible in the control or DKA exposure treatment groups. Subsequent MALDI-TOF-MS identification (Fig. S1) found 26 positive protein spots with 14 proteins up-regulated and 12 proteins down-regulated (Fig. 2).

To gain additional information on the differentially expressed proteins, the 26 proteins were further investigated using MALDI-TOF-MS. Confidence in the peptide mass fingerprinting matches (p<0.05) was based on MOWSE Scores greater than 58. The mass spectrum of the identified subset protein spots is shown in Table 1. COG function prediction classified the 26 positively expressed proteins into 13 functional categories (Fig. S3) with all proteins demonstrating high homology with the *Danio rerio* genome. A high proportion of gene functions were related to signal transduction mechanisms and cytoskeleton.

**Table 1:** The differential protein spots between control and DKA-exposed treatments by MALDI-TOF-MS.

Spot#	Accession NO.	Gene product	MW(kDa)	pI	Matched Peptides	Cov	Score	E-value	Fold change (Mean ± SD)	Location chromosome
2416	gi|57222259	Talin-1	272579	5.86	28	14%	68	0.006	−2.19±0.46	10
2411	gi|45387723	Uncharacterized protein LOC402952	37349	9.23	7	36%	58	0.055	−3.64±0.05	21
2327	gi|41944596	Heat shock protein 4	95585	5.10	17	24%	63	0.020	−2.34±0.71	14
6459	gi|326666090	Multidrug resistance-associated protein 1-like	228609	8.71	15	10%	64	0.014	−3.53±0.39	3
4407	gi|366039974	ATP synthase subunit beta, mitochondrial	55210	5.14	12	30%	76	0.001	−3.51±0.05	11
3502	gi|82181062	Beta-coat protein	108047	5.72	17	24%	62	0.023	−3.72±0.43	7
3730	gi|300680985	EF-hand calcium-binding domain-containing protein 4A	46004	5.82	18	39%	63	0.018	−3.15±0.56	25
1325	gi|18859049	Myosin light chain, phosphorylatable, fast skeletal muscle a	18967	4.68	11	72%	90	4.1e-05	−2.70±0.06	3
2203	gi|62202893	Mcm10 protein, partial	37666	5.48	11	32%	66	0.009	−3.40±0.56	4
2350	gi|18858249	Actin, alpha cardiac muscle 1	42288	5.23	12	43%	59	0.048	−6.95±0.22	17
7301	gi|42560193	Actin, cytoplasmic 1, N-terminally processed	42068	5.30	8	27%	66	0.009	−4.27±0.38	1
7309	gi|55925421	Uncharacterized protein LOC492776	27126	5.46	11	45%	63	0.019	−2.23±0.30	9
1561	gi|125838841	Acid sphingomyelinase-like phosphodiesterase 3b-like	52763	6.73	10	21%	67	0.008	3.76±0.33	16
1448	gi|353558878	Maternal embryonic leucine zipper kinase	77612	8.88	7	18%	59	0.050	3.77±0.05	1
3319	gi|28277651	Actin, alpha 1, skeletal muscle	42298	5.23	15	47%	72	0.003	5.02±0.13	17
4359	gi|313482836	Plasma membrane calcium ATPase 3a	130715	5.41	15	14%	69	0.005	2.10±0.40	8
5339	gi|161612176	LOC560422 protein, partial	34141	8.56	9	32%	58	0.062	5.98±0.32	2
5346	gi|292619409	Hypothetical protein LOC794259	291570	6.76	22	11%	61	0.031	5.27±0.41	12
2359	gi|62955473	Actin, gamma-enteric smooth muscle	42232	5.29	12	34%	74	0.002	6.85±0.09	1
0297	gi|61806478	Cyclin-H	37420	5.77	13	40%	71	0.003	5.72±0.36	5
02105	gi|120538416	LOC557463 protein, partial	42884	8.73	10	36%	73	0.002	3.79±0.61	13
3225	gi|161611678	Zgc:171522 protein	22889	9.04	9	38%	64	0.016	4.73±0.23	1
4228	gi|68398755	Megakaryocyte-associated tyrosine-protein kinase	50794	8.98	13	20%	61	0.033	2.53±0.06	22
2258	gi|41053317	Developmentally-regulated GTP-binding protein 1	40726	8.83	9	21%	65	0.013	4.44±0.64	10
8411	gi|68353786	Hypothetical protein LOC558776	54015	9.60	8	28%	63	0.019	2.50±0.25	1
7648	gi|326674120	Vacuolar protein sorting-associated protein 37B-like	34412	9.62	6	20%	62	0.026	3.18±0.42	15

**Note**: In “fold change” column, “+” indicates up-regulation, and “−” denotes down-regulation, respectively.

### qRT-PCR analysis of differentially expressed genes

By means of agarose gel electrophoresis determination, the extracted RNA from all treatments showed good integrity and high purity (Fig. S2). The OD_260_/OD_280_ values were in the range of 2.01–2.03 and the RNA concentrations were 217.1–599.5 ng µL^−1^ for the control and DKA-exposed groups. All of the OD_260_/OD_280_ values were greater than 2.0, indicating no contamination of RNA by proteins, salts, or carbohydrates. Subsequently, 26 genes were validated by qRT-PCR to confirm the consistency between their levels of transcription and translation.

**Figure 3:**
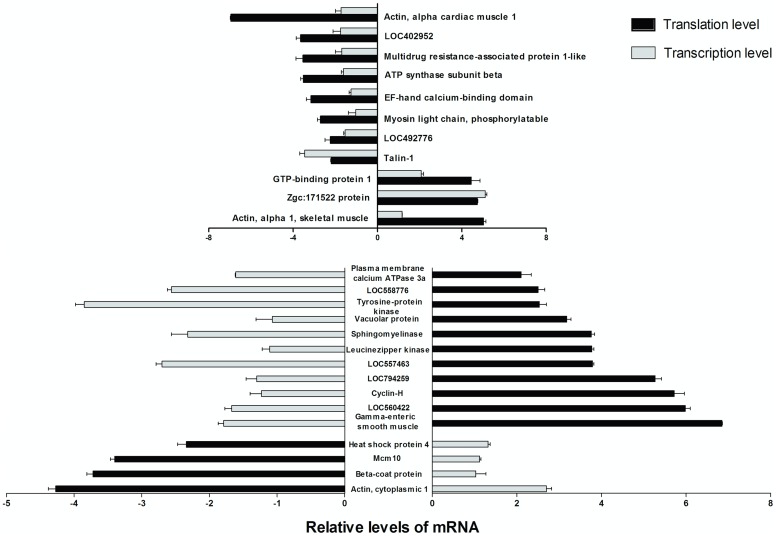
The relative mRNA transcription levels and translation levels of the positive proteins.

The relative mRNA transcription and translation levels for the positive proteins under DKA exposure are shown in Fig. 3. On the translational level, there were 14 genes with >2-fold up-regulation, and 12 genes with >2-fold down-regulation. In contrast, 8 genes were up-regulated while 18 genes were down-regulated on the transcriptional level. Of the 8 up-regulated genes, the genes with >2-fold transcriptional level were the sidekick cell adhesion molecule 1b (gi|161611678), the developmentally regulated GTP binding protein 1 (gi|41053317), and the heat shock protein 4b (gi|41944596). The down-regulated genes consisted of talin 1 (gi|57222259), lysine-rich nucleolar protein 1 (gi|68353786), acid sphingomyelinase-like phosphodiesterase 3b-like (gi|125838841), and megakaryocyte-associated tyrosine kinase (gi|68398755). Of the 26 target genes, 11 genes were consistent between their transcriptional and translational levels, while the other 15 genes were inconsistent. The 14 up-regulated gene functions were involved in signal transduction mechanisms, cytoskeleton and chaperones, while the 12 down-regulated genes involved signal transduction mechanisms, cytoskeleton, RNA processing and modification, energy production and conversion.

### Swimming behavior and locomotor activity of larvae

**Figure 4:**
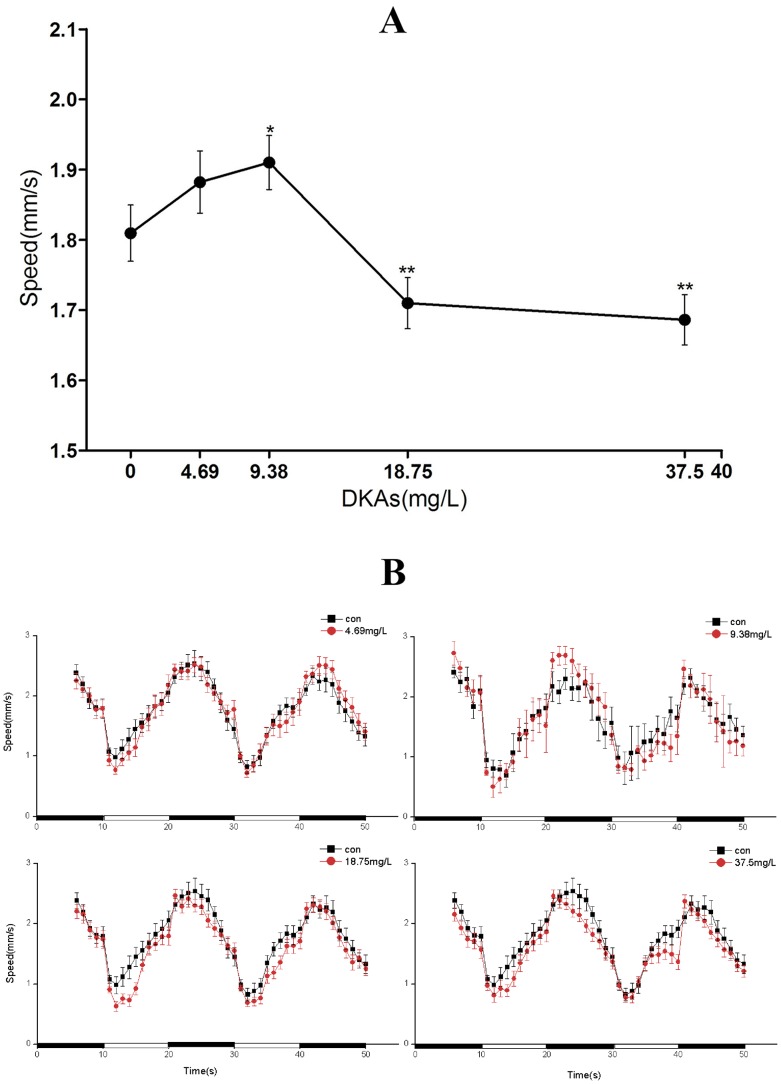
The swimming speed of larvae exposed to various DKA concentrations from 6 to 96(A) or a 50 min dark-to-light photoperiod (B) at 120 hpf.

Based on proteomic results, we determined that the functions of the positive proteins were mainly concerned with skeletal muscle, cardiac muscle, signal transduction, and energy transfer. Therefore, we surveyed the locomotor activity of zebrafish at 120 hpf (Figs. 4A and 4B) using two metrics; one examining the average swimming speed at 120 hpf and the other assessing the light-to-dark stimulation response of zebrafish. A prerequisite of these locomotor activity studies was not having visible malformation of larvae during the experimental process. The average swimming speed for the 4.69 and 9.38-mg/L DKA treatments were greater than the control group, while speeds were distinctly slower for the higher DKA treatments (18.75 and 37.5 mg/L) than the control group. The highest swimming speed was observed in the 9.38 mg/L DKA treatment (Fig. 4A). During photoperiod stimulation, the locomotor activity had a cyclical change for the control group. After an adaptation period from dark (5 min) to light, the swimming speed of larvae gradually increased. However, the locomotor activity suddenly increased to a peak value when the dark period was proceeded by 10 minutes of light. The locomotor activity of larvae in the light period was significantly lower than in the dark over the whole cycle. After DKA exposure, a prominent change of periodical locomotor activity for larvae occurred with photoperiodical stimulation. The swimming speed in the light for the 4.69 and 9.38 mg/L DKA treatments were similar to the control group. Under light stimulation, the larvae swimming speed was higher than the control group for the 9.38 mg/L DKA light treatment. However, the opposite response was observed for the 18.75 and 37.5 mg/L DKA treatments regardless of light or dark conditions (Fig. 4B).

### CK activity and Cr concentration assays

**Figure 5:**
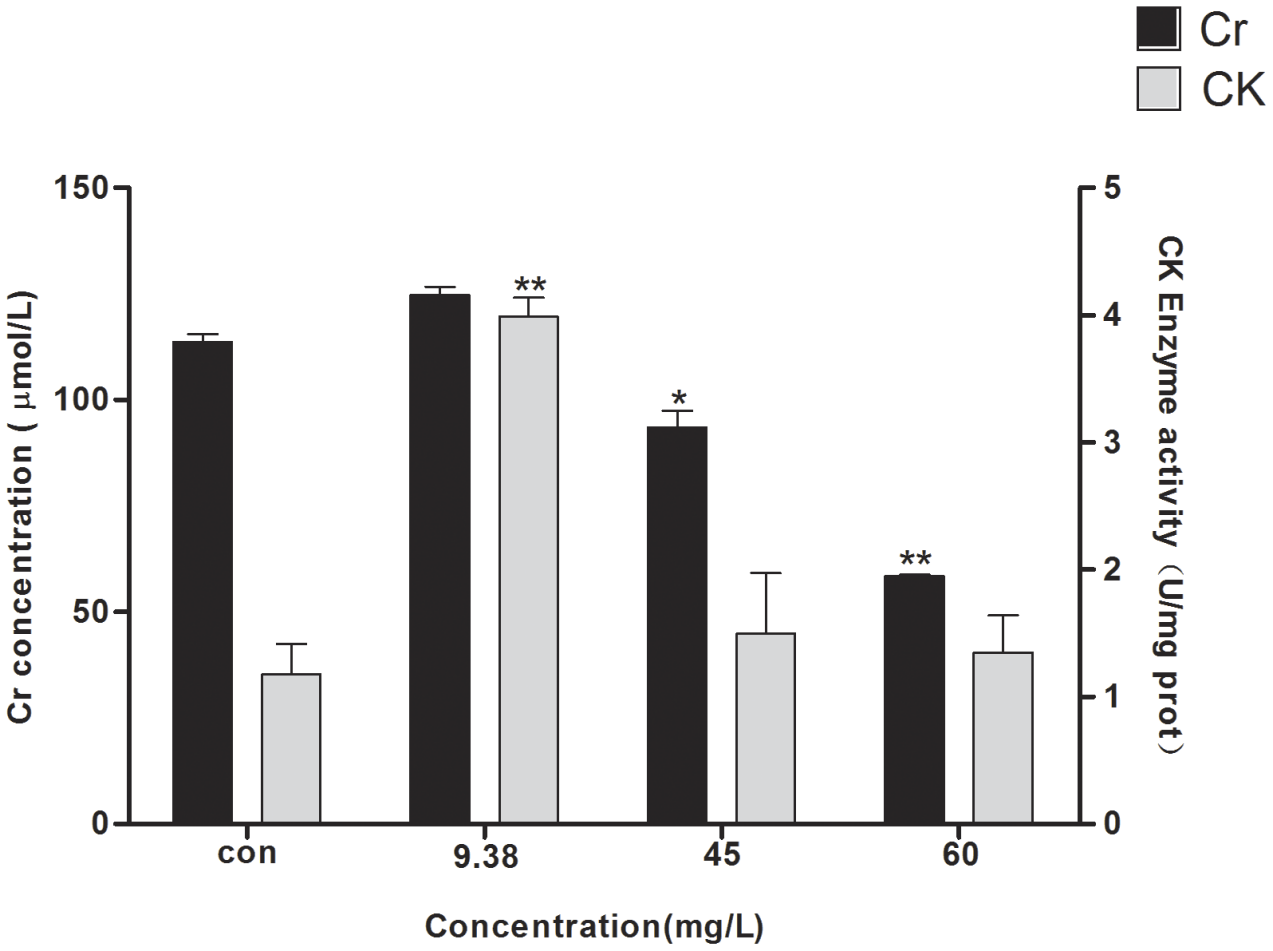
The effects of DKA concentrations on CK activities and Cr concentrations.

Zebrafish and mammals have high similarity in Cr synthesis and transport mechanisms. The Cr/phosphocreatine/CK system is one of the most important mechanisms for supplying energy in vertebrates. Zebrafish locomotor behavior is a direct result of nerve conduction, muscle contraction, and energy transfer. The above behavioral study demonstrated that DKAs had an effect on the locomotor activity and pattern of zebrafish in the photoperiod stimulation. In this study, CK activity and Cr concentration were determined when zebrafish were exposed to a series of DKA concentrations (9.38, 45 and 60 mg/L). For the 9.38-mg/L DKA treatment, CK activity was significantly increased by more than 2-fold, but change in Cr concentration was observed relative to the control. However, CK activity and Cr concentration were sharply decreased in the 45 and 60-mg/L DKA treatments when compared to the 9.38 mg/L DKA treatment (Fig. 5). The changing trend for both CK activity and Cr concentration were similar to the trends for locomotor activity (Fig. 4).

### Cardiac development and heart rate analysis

**Figure 6:**
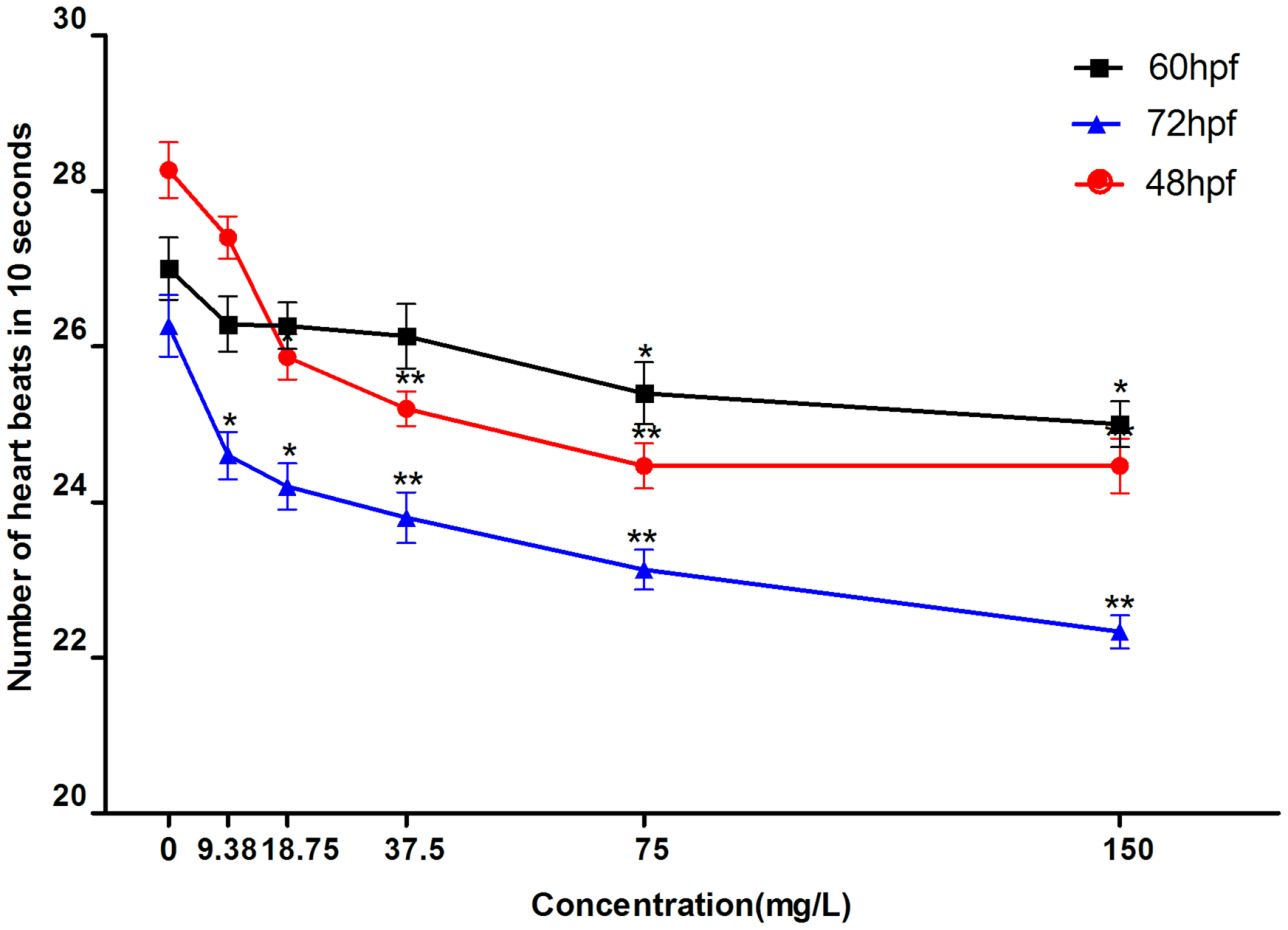
The heart rate of zebrafish under different DKA concentrations.

If cardiac development is influenced by DKA exposure, the location of the atrium and ventricle along with the distance between the sinus venosus to bulbus arteriosus (SV-BA) will be altered accordingly. There was no obvious change in SV-BA after DKA exposure (data not shown), indicating that DKAs had no significant influence on cardiac development of zebrafish over the investigated DKA concentration range (9.38–150 mg/L). Because the cardiovascular system development of zebrafish terminates at 48 hpf, we measured the heart rate at 48, 60, and 72 hpf. The embryonic heart rates under DKA exposure were significantly reduced compared to the control group (Fig. 6). The heart rates of zebrafish for the control and 9.38 mg/L DKA treatment were remarkable higher (*p*<0.01) at 48 hpf than those at 60 and 72 hpf. For 60-hpf larvae, higher heart rates were observed than for 48 and 72-hpf larvae at the same DKA concentrations (18.75, 37.5, 75, and 150 mg/L). Obviously, the changes in heart rate had a dose-dependent relation, i.e., a reduction of heart rate concomitant with an increase in DKA concentrations (Fig. 6).

### Histopathological analysis

**Figure 7:**
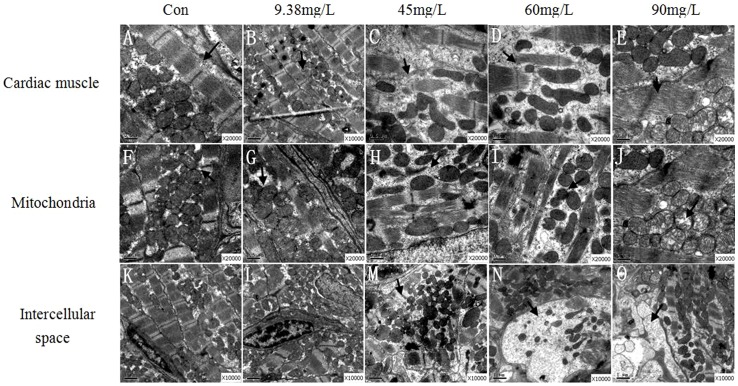
Histopathological analysis of heart tissue.

Histopathological observation of zebrafish cardiac tissue was conducted by transmission electron microscope across a series of DKA concentrations (9.38, 45, 60 and 90 mg/L). DKA exposure induced severe histopathological changes in zebrafish cardiac tissue, reflected in the changes of muscle fibers and mitochondria (Fig. 7). The normal myocardial fibers can be seen clearly in the control group (Fig. 7A), while fracture, chaos, and even partial dislocation occurred in DKA-exposed treatments (Fig. 7C–E). As for the mitochondria, normal mitochondria organelles were complete in the control group, while they were closely crowded around the myofibril in DKA-exposed treatments (Fig. 7F). In the 45 and 60 mg/L DKA treatments, contrasting changes in mitochondria were observed, such as dispersive arrangement, irregular shape, and slight deformation (Fig. 7G–H). In the 90-mg/L DKA treatment, partial dissolution of cristae and vacuolation of mitochondria were also observed (Fig. 7J). Furthermore, the cells were obviously swelling in DKA-exposed treatments (Fig. 7M–O) as compared to the control group (Fig. 7K). However, no obvious changes of mitochondria and cardiac muscle were observed for the 9.38 mg/L DKA treatment compared with the control.

## Discussion

DKAs are detected in many environmental matrices where they create a “pseudo-persistent” phenomenon due to their large dosages and frequent application [2], [3]. Although trace level DKA exposure does not cause direct and acute effects, long-term exposure in aquatic ecosystem leads to chronic toxicological effects on aquatic organism and human health [5], [6]. The presence of many DKAs coexisting in the environment poses an especially large threat to organisms throughout the environment. To address this concern, this research examined the effects of mixed DKA exposure to zebrafish at trace or sublethal doses common in many environments. Using recent advances in omics technology, we studied differentially expressed proteins resulting from long-term DKA exposure. We verified the proteomic experimental results using several molecular techniques (qRT-PCR, behavioristics, biomarkers and histopathology) to demonstrate the toxic effects of DKAs under low-dose, long-term exposure. These results provide the basis for the diagnosis and treatment of medicine source disease.

Although concentrations of single DKA species are often detected at µg/L levels in environmental waters, they can reach mg/L levels in receiving waters from some specific locales, such as hospitals and pharmaceutical factories. Hospital wastewater generally contains DKA concentrations in the µg/L to occasionally mg/L levels [20]. In wastewaters from a German hospital, ciprofloxacin was detected at concentrations up to 124.5 µg/L in 24 samples [21]. Multiple classes of antibiotics with relatively high concentrations, commonly at >100 µg/L levels, were detected in swine waste storage lagoons in the United States [18]. Similarly, Ben et al. [19] detected three tetracyclines in swine wastewater with concentrations up to 32.7 µg/L. It is important to note that the previously reported concentrations refer to a single diketone antibiotic, and that the combined concentrations of mixed DKAs can be much higher than mg/L levels. Therefore, the higher concentrations of mixed DKAs used in this study (9.38 to 60 mg/L with each DKA concentration ranging from 1.56 to 10 mg/L) conservatively represent the worst case scenario for direct exposure to hospital or farm-derived animal wastewater with no dilution.

Cell signal transduction refers to an extracellular signal stimulating a cell through cell membrane or intracellular receptors. Changes in signal transduction can affect cell growth, proliferation and metabolism, and even induce cell death [22]. Development-regulated GTP-binding proteins (DRGs) are a subclass of G proteins, which contain five structural motifs (G1–G5) that are conserved in all GTP-binding proteins [23]. In the mouse skeletal muscle, heart and kidney, high levels of DRGs have been observed [24]–[25]. Wei *et al.* [23] demonstrated that DRG was ubiquitous at the cleavage stage, blastula stage and segmentation stage of zebrafish, and that expression of DRG was concentrated in the eye, head and trunk region. Brand and coworkers found three mutagenesis screens in zebrafish-*acerebellar(ace)*, *no isthmus(noi)*, *spiel ohnegretzen(spg)* by means of massively artificial mutation [26]–[28]. Several genes, like the *En, Pax, Otx* and *Gbx* families, are very important in the development of the nervous system, but there is not a clear role concerning the interactive relationship among them in formation of the midbrain-hindbrain boundary (MHB). Three signaling pathways, involving *pax2.1*, *wnt1* and *fgf8*, are activated independently in early anterior-posterior patterning of this area. Mutations in the zebrafish *no isthmus (noi)* gene alter development of the MHB, and affect the *pax2.1*gene (formerly *pax(zf-b)*) [27]. *Fgf8* and *pax2.1* are activated in adjacent domains that only later become overlapping, and activation of *Fgf8* occurs normally in no isthmus embryos that are mutant for *pax2.1* [28]. In zebrafish, an interaction between *otx2* and *gbx1* determines the site of MHB development, and *gbx1* can partially restore hindbrain patterning in cases of *Wnt8* loss-of-function [29]. As a consequence, regulatory relationships between these genes are very intricate, and future research is warranted to better understand the formation of MHB. *Drg1* possibly regulates these genes and controls midbrain-hindbrain differentiation that acts as one of the control factors and signaling proteins. In this investigation, *Drg1* was up-regulated, and its over-expression led to the signal transduction disorder and further affected formation of the midbrain, hindbrain and cerebellum. As a result, the nervous system of zebrafish was severely damaged, which resulted in myodystonia and behavior disorders.

Based on our results, some of the zebrafish genes showing inconsistency between transcriptional and translational levels perform the primary regulating role. Regulation of gene expression can be controlled by post-transcriptional processing, mRNA degradation, translation and post-translational processing, protein degradation or other aspects. The degradation of mRNA transcripts is a possible reason for the inconsistency between transcriptional and translational levels. Some inducible gene transcripts can be degraded immediately after translation and even in the course of translation [30]. The weak correlation between transcription and protein levels may result from many factors, and thus further investigation is required to elucidate specific mechanisms.

Biomarkers are signal indicators that can form in response to environmental pollutants, and thus they are widely used for ecological risk assessment in aqueous environments. CK is an important kinase, directly related to energy operations in cell, muscle contraction and ATP regeneration. It catalyzes the reversible transfer of high energy phosphate from ATP to creatine, and further facilitates storage of energy in the form of phosphocreatine, which ensures energy requirements for cell tissues and cellular physiological activities. Because muscle cells are rich in CK, the CK levels in the supernatant of tissue will be elevated when muscle fibers are destroyed and it will lead to the release of large amounts of CK into the blood [23]. Serum Cr concentration is proportional to the amount of exercise, and is also regarded as an important indicator for injured glomerular filtration function because a certain quantity of Cr can't be absorbed by the nephridial tubule after filtration. At lesions in kidneys, the level of serum Cr will increase due to Cr blockage of excretion, and thus it is an important indicator to assess renal function. In the 9.38-mg/L DKA treatment, a sudden increase of CK activity and Cr concentration was observed concomitantly with accelerated zebrafish speed (Fig. 5A). In contrast, CK activities were substantially decreased in the 45 and 60 mg/L DKA treatments when compared with the 9.38 mg/L DKA treatment. Decreased zebrafish speed resulted in less energy demand, which further affected the CK activity (one of the important energy indicators). The levels of Cr in zebrafish serum in high-dose DKA treatment exposure (45 and 60 mg/L) were significantly lower than those of the control group, suggesting a significant inhibiting effect of DKAs on zebrafish locomotor activity. This inhibition of locomotor activity decreases the energy requirement of zebrafish by reducing Cr concentration in the blood through metabolism, which was consistent with the results of the behavioral study.

Heart rate is the most sensitive index to evaluate cardiac function. The development process of the zebrafish heart has a similar gene-regulating pathway to that of humans. The heart valve of zebrafish forms at 48 hpf, and a perfect cardiovascular circulation system forms at 72 hpf. If cardiac development is affected, the location of the atrium and ventricle will alter accordingly resulting in difference in the SV-BA distance, which is an important index of cardiac cyclization degree. As a result, the effect of drugs on the heart can be quantified by measuring the SV-BA distance [31]–[32]. Because there are many factors that can lead to decreasing zebrafish heart rate, the specific mechanism responsible for this change is not clearly known. In this experiment, the damaged myocardial cells affect the scalability of the heart, which finally result in slowing of the heart rate. It was first reported by Knorr *et al.* [33] that ciprofloxacin could induce the prolongation of the Q-T interval. Later, many fluoroquinolone antibiotics were shown to prolong, the Q-T interval [34], which triggered an arrhythmia.

Changing behavior is a comprehensive response to external and internal environmental changes. At an individual level, changes in biological behavior can reflect toxicological effects of environmental pollution on biological metabolism and the functions of nerve, muscle and organs. Jin *et al.* [35] found that the insecticide bifenthrin increased the spontaneous movement frequency by prolonging repetitive action potential due to channel opening. In this research, the larvae exposed to 4.69 or 9.38-mg/L of DKAs had a higher swimming speed, suggesting that the lower DKA concentrations had an incentive effect on larval motor neuron function. Under conditions of light-to-dark stimulation, zebrafish behavior showed a regular biorhythm [36], i.e., first active after exposed to sudden darkness and then gradually becoming inactive. Based on the experimental results of the differentially expressed proteins, we speculated that the declining swimming ability of zebrafish may be due to abnormal muscle and innervation.

## Supporting Information

Figure S1 **2-DE and the mass spectrum of protein spots in the control and 9.38 mg/L treatment.**
(TIF)

Figure S2 **The profile of total RNA in control and 9.38 mg/L DKA-exposed treatments.**
(TIF)

Figure S3 **COG functional classification of 26 positively differential expression proteins.**
(TIF)

Table S1 **The primers used for amplification of target and β-actin protein genes.**
(DOC)
