# Supplementary figures and images for: Toxicological Assessment of Trace β-Diketone Antibiotic Mixtures on Zebrafish (Danio rerio) by Proteomic Analysis
Source: PLoS One. 2014 Jul 25;9(7):e102731. doi: 10.1371/journal.pone.0102731 (PMC4111491; doi:10.1371/journal.pone.0102731)

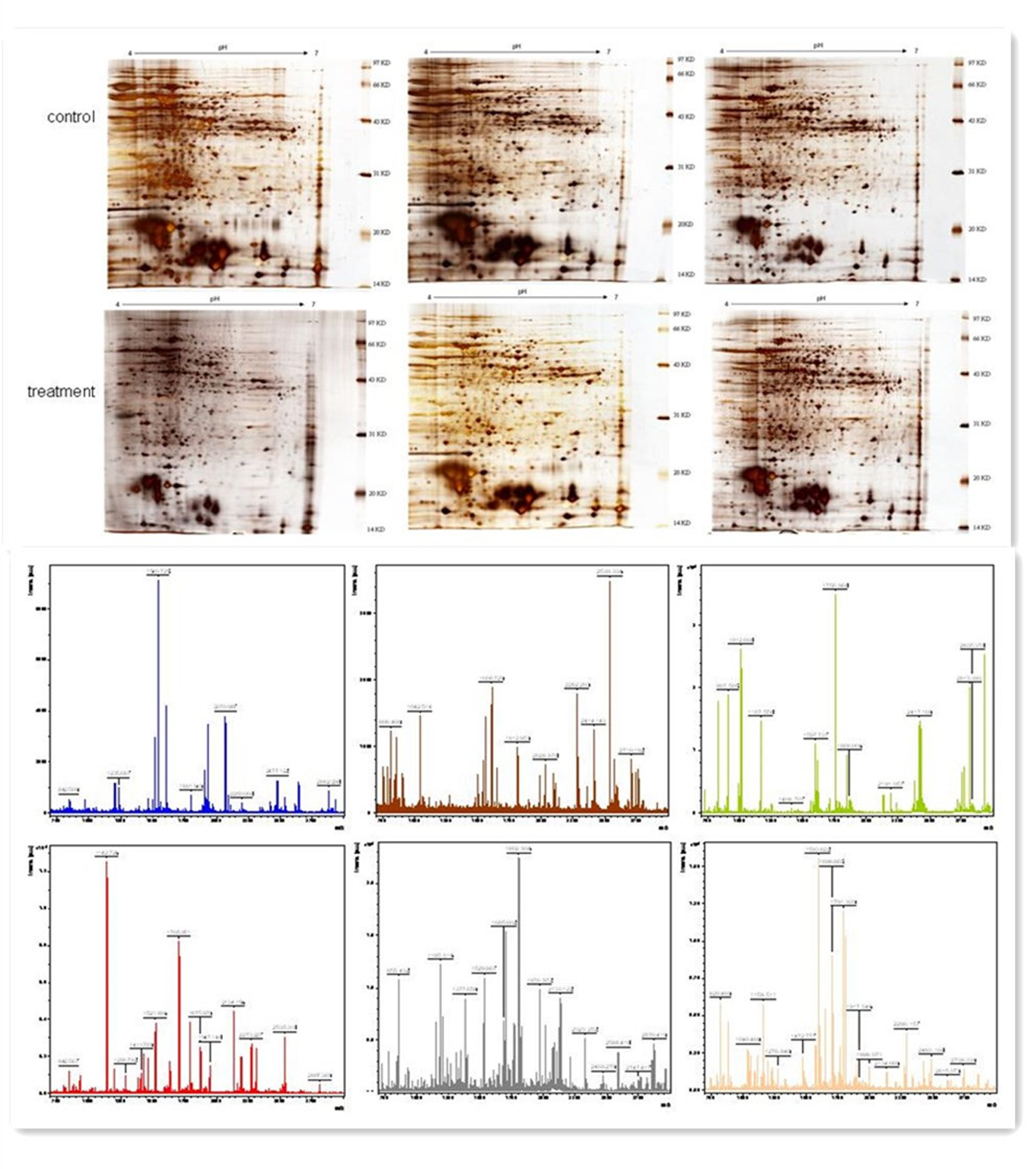

Supplement: Figure S1 — 2-DE and the mass spectrum of protein spots in the control and 9.38 mg/L treatment. (TIF) [file pone.0102731.s001.tif]

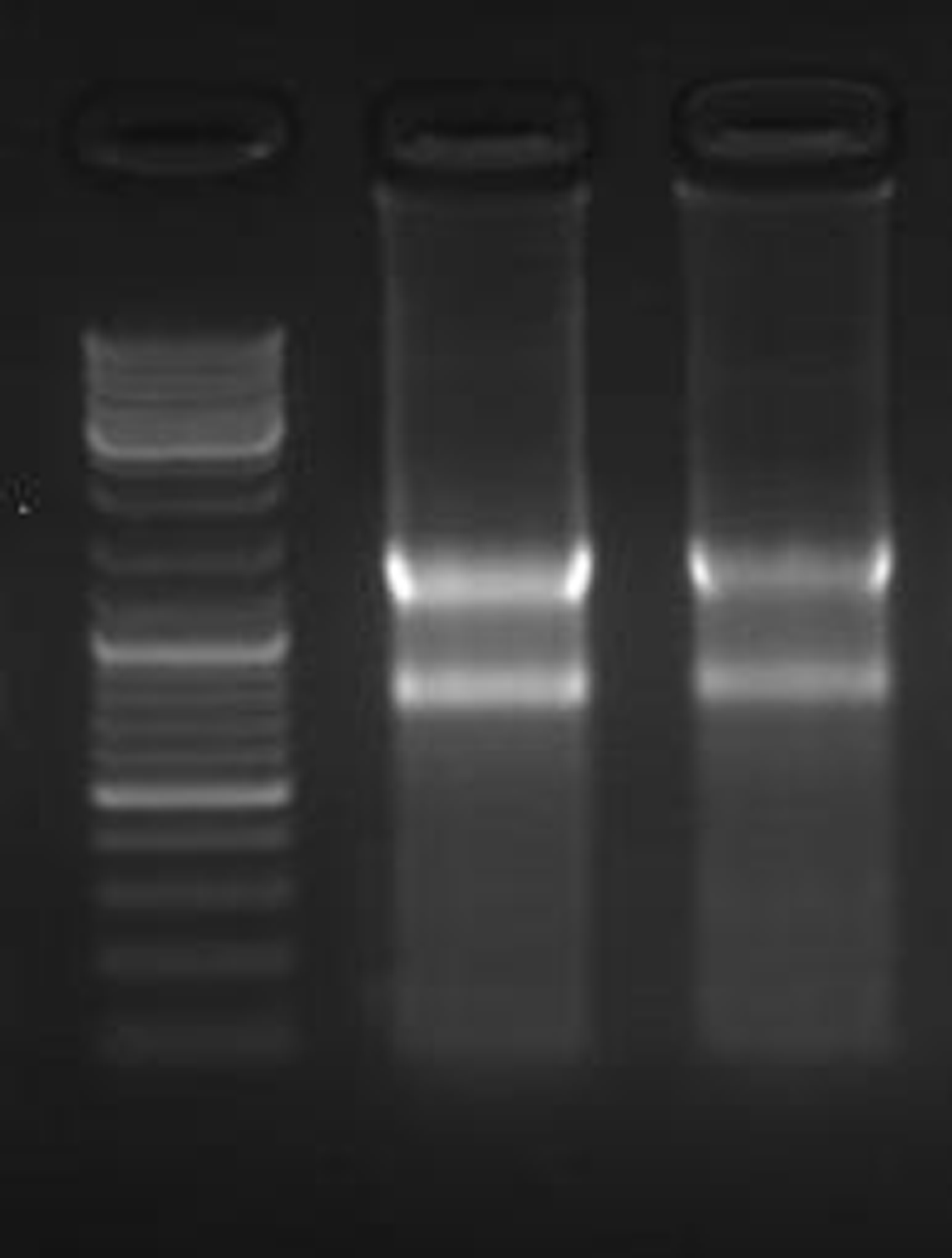

Supplement: Figure S2 — The profile of total RNA in control and 9.38 mg/L DKA-exposed treatments. (TIF) [file pone.0102731.s002.tif]

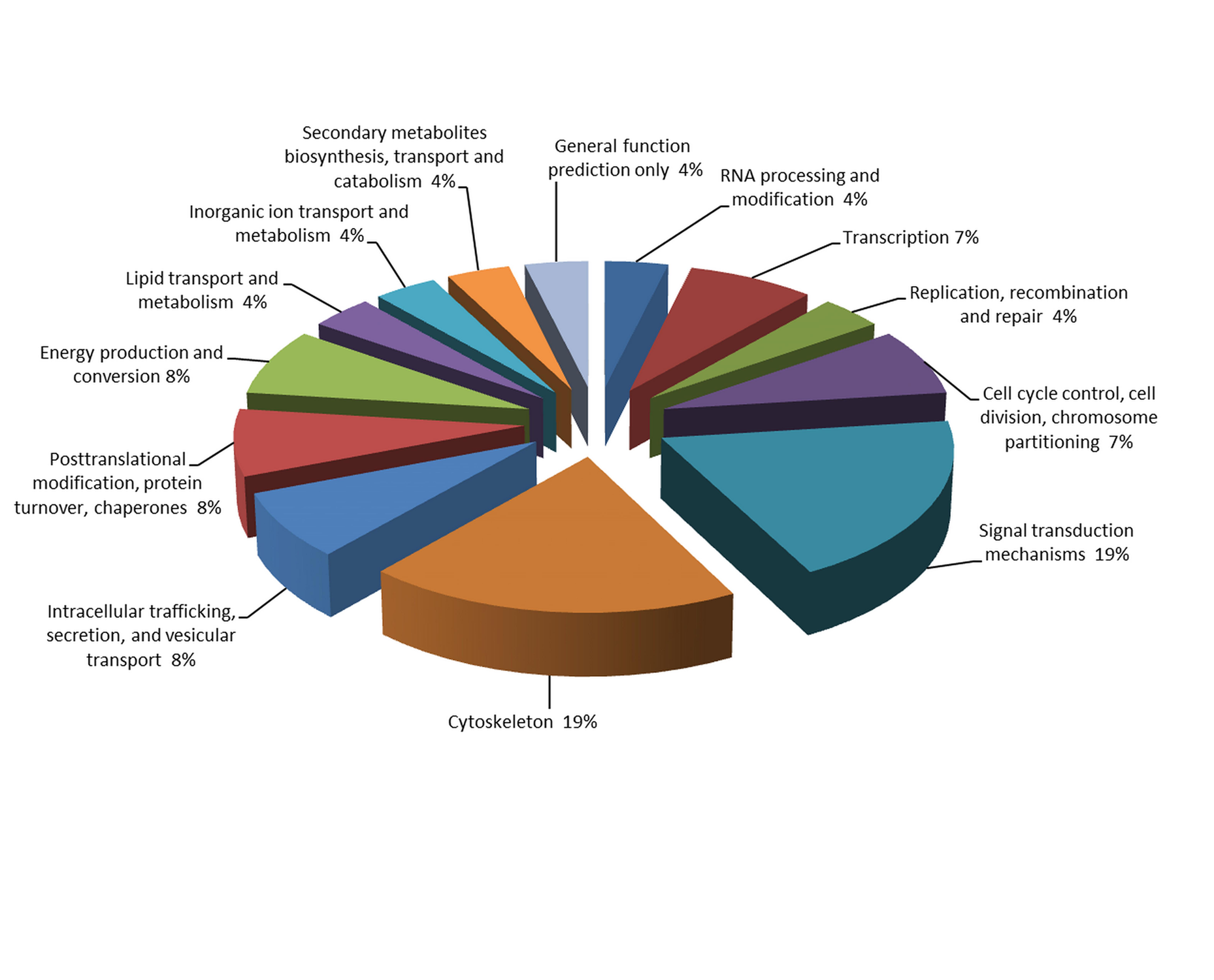

Supplement: Figure S3 — COG functional classification of 26 positively differential expression proteins. (TIF) [file pone.0102731.s003.tif]
