# Supplementary material for: Toxicological Assessment of Trace β-Diketone Antibiotic Mixtures on Zebrafish (Danio rerio) by Proteomic Analysis
Source: PLoS One. 2014 Jul 25;9(7):e102731. doi: 10.1371/journal.pone.0102731 (PMC4111491; doi:10.1371/journal.pone.0102731)
Supplement: Table S1 — The primers used for amplification of target and β-actin protein genes. (DOC) [file pone.0102731.s004.doc]

**Table S1.**

| Gene name | Forward primer sequence | Reverse primer sequence | Amplified fragment length |
| --- | --- | --- | --- |
| *acta1a* | TGAGGAGCACCCAACACT | TGCGGTCAGCAATACCAG | 644bp |
| *acta1b* | CGAGCACGGCATCATTAC | GCTGTTGTAGGCGGTTTC | 631bp |
| *aclyb* | TGTTGTCGCAATGGTCTA | CTTGATGCCTGGAGTGTC | 559bp |
| *atp5b* | GACCCGTGAGGGAAACGA | TGGTGGCATCCAAGTGAG | 431bp |
| *atp2b3a* | ACTGCAAGAAAGGGAAAC | AAATGACGCACAAGGTTA | 466bp |
| *copb1* | CCAATGACTCCGAACCAC | CCGAAAGTATGCACCTGA | 616bp |
| *ccnh* | ATTAGGGACGTGGGTTAA | GTCATTGTTGCTCGGTTG | 622bp |
| *drg1* | AGAAAGACAAGGGCGGAATC | CATGCAAGGCATCGGAGT | 579bp |
| *efcab4a* | CTGGACAGTATGAGGCAAGG | GGGCGTGAAGGAGTGTTT | 441bp |
| *hspa4b* | GAAATCGGTCTATTGGAG | GTTTGGTCTTCGCATCTA | 648bp |
| *knop1* | GGTGGAGGACGGAGTGAT | CGCAGGAACTTGGCTTGT | 639bp |
| *melk* | CCCCTGATTGCTACGATG | ACTTTCCCTCCCTTACGC | 517bp |
| *matk* | GCAGACAACCGTATCCCA | ATTCACCCAATCAACCAA | 597bp |
| *mcm10* | GGCAGATTCCTATTGGTC | CAGAACTGGCATTCATACA | 649bp |
| *mylz2* | ACTCAGTGCGACAGGTTC | ACTCCATCGTGCTTCTTT | 612bp |
| *sdk1b* | GACACCGCTCCGTACTTT | AACCACGATTCCTCTTGTTTAT | 568bp |
| *tln1* | GAGGACGAGGGCGGAAAT | TCCAGGATGCGGTCGGTA | 629bp |
| *zgc:77262* | GACCAGTGGGAAGGAACA | TACGCAAACAATAGGTAAACAG | 417bp |
| *Zgc:101559* | AATAAAGGAACTAAGCCGACAA | ACGCCAAATATCCAAGACAAAG | 402bp |
| *LOC560422* | TCACTCAACCGCTCTATC | GCTAAGCCCAATGTCTCC | 586bp |
| *LOC564392* | TGTCTGCGACTCACCCTG | TGATGCCCGAAGAACTGG | 645bp |
| *LOC100334180* | CCTAAGCTGGATTGTGCG | AACTGGGTTCGGATGGTG | 699bp |
| *si:ch211-227c6.* | GCGGTGTTGGACAGTTTA | GGGCTAGGGTTGGTTATG | 413bp |
| *gamt* | CAGCAGTCCACCATGAGC | CTGTGAGCGTGAGCCTTG | 479bp |
| *zgc:86709* | AATGCGTTTGGATTTGGC | CTGGAAGGTGGACAGGGA | 535bp |
| *paics* | TGGTATCAACCCAGAGTGC | AGGAGTGAAGCGGTAGCC | 529bp |
| *β-actin* | AGCCTTCCTTCCTGGGTA | AGGGCAAAGTGGTAAACG | 575bp |
